# A risk prediction system of postoperative hemorrhage following laparoscopy-assisted radical gastrectomy with D2 lymphadenectomy for primary gastric cancer

**DOI:** 10.18632/oncotarget.20828

**Published:** 2017-09-11

**Authors:** Xin-Sheng Xie, Jian-Xian Lin, Ping Li, Jian-Wei Xie, Jia-Bin Wang, Jun Lu, Qi-Yue Chen, Long-Long Cao, Mi Lin, Ru-Hong Tu, Chang-Ming Huang, Chao-Hui Zheng

**Affiliations:** ^1^ Department of Gastric Surgery, Fujian Medical University Union Hospital, Fuzhou, Fujian Province, China; ^2^ Department of General Surgery, Fujian Medical University Union Hospital, Fuzhou, Fujian Province, China; ^3^ Key Laboratory of Ministry of Education of Gastrointestinal Cancer, Fujian Medical University, Fuzhou, Fujian Province, China; ^4^ Fujian Key Laboratory of Tumor Microbiology, Fujian Medical University, Fuzhou, Fujian Province, China

**Keywords:** gastric cancer, postoperative hemorrhage, laparoscopy, D2 lymphadenectomy, risk factor

## Abstract

**Objectives:**

To investigate risk factors of postoperative hemorrhage (PH) following laparoscopy-assisted radical gastrectomy (LARG) with D2 lymphadenectomy for primary gastric cancer (PGC) and to use those risk factors to develop a scoring system for risk assessment.

**Materials and Methods:**

A total of 1789 PGC patients were enrolled in our study. We analyzed the risk factors of PH and constructed a scoring system using 75% of the cases as the experimental group and 25% of the cases as a verification group to demonstrate the effectiveness.

**Results:**

Among these 1789 patients, 46 (2.6%) developed PH. Univariate and multivariate analysis in the experimental group indicated that having more than 41 lymph node excisions, combined organ resection, stage III tumor and postoperative digestive fistula were independent risk factors of PH. According to the independent risk factors, we constructed a scoring system to separate patients into low-risk (0–2 points) and high-risk (≥ 3 points) groups. The area under the ROC curve for this scoring system was 0.748. In the verification group, the risk of PH predicted by the scoring system was not significantly different from the actual incidence observed.

**Conclusions:**

This scoring system could simply and effectively predict the occurrence of PH following LARG with D2 lymphadenectomy for PGC. The predictive system will help surgeons evaluate risk and select risk-adapted interventions to improve surgical safety.

## INTRODUCTION

Although the morbidity and mortality of the primary gastric cancer has declined in recent decades, it is still the second most common cause of cancer-related death worldwide [1–3]. Surgery remains the most important intervention for primary gastric cancer (PGC), and radical gastrectomy with D2 lymphadenectomy has become the standard procedure [4, 5]. Even with recent developments in surgical technology, postoperative complications are still difficult to avoid. Compared with the postoperative anastomotic stenosis and gastric paralysis, postoperative complications such as chylous fistula and postoperative hemorrhage (PH) often occur under emergency conditions and endanger the patient's life [6, 7]. If we can identify the risk factors of PH following gastric cancer (GC), we could perform a risk assessment and determine appropriate perioperative interventions which would be helpful in reducing the mortality of PH following GC.

Research concerning the risk of PH following laparoscopy-assisted radical gastrectomy (LARG) is very limited. Therefore, this study analyzed the risk factors as they relate to the incidences of PH in 1789 patients who underwent LARG with D2 lymphadenectomy in our department. We also established a new, simple and practical scoring system to help surgeons assess and predict the risk of PH following gastrectomy.

## MATERIALS AND METHODS

### Materials

This study was a retrospective analysis of a prospectively collected database of 1789 PGC patients treated with LARG with D2 lymphadenectomy in the Department of Gastric Surgery of Fujian Medical University Union Hospital, Fuzhou, China, between May 2007 and September 2013. All the clinicopathologic data of patients were recorded in a clinical data system for gastric cancer surgery. The tumor stage was determined based on the 8th edition (2016) of the International Union against Cancer (UICC) tumor, lymph node and distant metastasis (TNM) classification [8, 9]. Patients with intraoperative evidence of peritoneal dissemination, invasion of the adjacent organs, suffer from coagulation disorders or a distant metastasis, conversion to an open laparotomy or incomplete pathological data were not included in this study. All patients voluntarily chose laparoscopic surgery, and signed an informed consent following an explanation of the surgical and oncological risks. The type of surgical resection and the extent of lymphadenectomy were selected according to the 4th edition Japan stomach cancer treatment guidelines [4]. {Association1, 2016 #1}All LARG procedures were carried out by the same group of surgeons.

### Variables and definitions

The definition of PH, based on the literature, is blood flow out of the surgical site with a drop in hemoglobin of more than 2 g/dl in 24 hours usually accompanied by hemorrhage and requiring at least 2 units of packed red blood cells [10–12]. The diagnostic procedures of PH were considered as follow: clinical manifestation, blood routine, blood pressure measurement, bedside ultrasonography, abdominocentesis, or angiography and so on. We considered every 24 hours after surgery as one day and defined the day of diagnosis when patients showed the signs of bleeding or bleeding conformed by auxiliary examinations. The potential risk factors for PH were extracted from the database. These included gender, age, body mass index (BMI), previous abdominal surgery, tumor location, tumor size, TNM stage, operative time (recorded from the skin incision to skin closure), type of surgical resection, type of reconstruction, blood loss during the operation, number of resected lymph nodes, combined organ resection, digestive tract fistula, pure abdominal infection and chylous fistula.

### Statistical analysis

All statistical analyses were performed using the Statistical Package for Social Science (SPSS) version 23.0 for Windows (IBM, Chicago, IL, USA). The results were expressed as percentages or as the means ± standard deviation (SD) unless otherwise noted. Data were analyzed using the chi-squared test, Fisher's exact test or student's *t*-test. Risk factors for PH were assessed by univariate and multivariate analyses using a logistic regression model. The variables with *P* < 0.05 in the univariate analysis were subsequently transformed into appropriate binary classification variables and included in a multivariate binary logistic regression model. The data from the multivariate analysis and the grouping of each risk are shown as OR values with corresponding 95% confidence intervals. The receiver operating characteristic (ROC) curve was calculated to assess the reliability of this model to distinguish between patients with and without PH. The area under the curve (AUC) was also measured, shown as the absolute value and 95% confidence interval (95% CI). An area under the curve of 0.7 or above was considered clinically significant. To build a scoring system to predict risk, patients were assigned random numbers and sorted in numerical order. The 75% of patients with the largest numbers were assigned to the experimental group; the others were assigned to the validation group. The validated prediction system categorized patients as high-risk or low-risk according to their score. We verified the accuracy of the prediction system by comparing the difference between the incidence of prediction and the actual incidence of PH. *P* values < 0.05 were considered statistically significant.

## RESULTS

### The occurrence of PH and the clinicopathological factors between the experimental and verification group

There were 1789 cases of patients who underwent LARG with D2 lymphadenectomy for primary gastric cancer. Among them, 46 (2.6%) suffered from PH. Of the 46, 37 (80.4%) were male and 9 (19.6%) were female; the mean age and BMI were 60.56 ± 11.14 years and 22.18 ± 2.96 kg/m^2^, respectively. On average, hemorrhage usually occurred in the fifth day after surgery. The clinicopathological factors of the two groups are shown in Table 1.

**Table 1:** The clinicopathological factors between the experimental group and the verification group

Variables	Experimental group (*n* = 1342)		Verification group (*n* = 447)	
	Hemorrhage(*n* = 34)	No hemorrhage (*n* = 1308)	Hemorrhage (*n* = 12)	No hemorrhage (*n* = 435)
**Gender**	±			
Male	27	998	10	308
Female	7	310	2	127
**Age(years)**	63.06 ± 12.79	60.43 ± 11.13	62.08 ± 16.08	60.73 ± 10.88
**BMI (kg/m^2^)**	21.47 ± 2.18	22.19 ± 3.01	22.1 ± 2.22	22.2 ± 2.88
**PAS**				
Yes	6	176	1	68
No	28	1132	11	367
**Tumor size (cm)**	48.78 ± 19.29	46.27 ± 25.27	52.08 ± 25.54	46.84 ± 25.77
**Tumor location**				
Upper	13	304	6	117
Middle	2	210	1	64
Lower	14	656	3	206
Diffuse	5	138	2	48
**TNM stage**				
I/ II	6	627	5	218
III	28	681	7	217
**Operation time (min)**	199.65 ± 62.75	190.19 ± 61.72	240.00 ± 94.89	196.48 ± 67.45
**BLDO(ml)**	118.24 ± 143.43	85.73 ± 103.39	100.50 ± 80.52	84.83 ± 86.88
**Resected LNs (*n***)	32.13 ± 10.27	33.91 ± 13.15	31.83 ± 7.03	34.71 ± 13.90
**Operation type**				
Total	19	623	9	227
Subtotal	15	685	3	208
**Type of Anastomosis**				
Roux-en-Y	19	622	1	11
B-I	9	538	2	156
B-II	5	120	0	41
Esophagogastric	1	28	9	227
**Combined resection**				
Yes	9	98	5	32
No	25	1210	7	403
**DTF**				
Yes	6	36	3	5
No	28	1272	9	430
**PAI**				
Yes	3	39	0	12
No	31	1269	12	423
**Chylous fistula**				
Yes	3	42	1	15
No	31	1266	11	420
**Hospital stay (days)**	20.09 ± 9.67	13.86 ± 7.64	19.92 ± 7.28	14.36 ± 9.29

PAS: Previous abdominal surgery; PAI: Pure abdominal infection; DTF: Digestive tract fistula; BLDO: blood loss during the operation.

### Univariate analysis of PH in the experimental group

The univariate analysis of PH in the experimental group indicated that tumor stage (*P* < 0.001), number of lymph nodes excised (*P* < 0.05), combined organ resection (*P* < 0.001) and postoperative digestive tract fistula (*P* < 0.001) were related to PH, with the differences showing statistical significance. However, gender, age, BMI, previous abdominal surgery, tumor size, tumor location, operative time, blood loss during the operation, operation and anastomotic methods, pure abdominal infection and chylous fistula had no effect on PH (*P* > 0.05). See Table 2.

**Table 2:** Univariate analysis of PH

Variables	Early Hemorrhage (*n* = 34)	Later Hemorrhage (*n* = 34)	No Hemorrhage (*n* = 1308)	*P* value
**Gender**				0.673
Male	27		998	
Female	7		310	
**Age(years)**				0.192
≤ 65	16		473	
< 65	18		835	
**BMI (kg/m^2^)**				0.093
≥ 25	3		269	
< 25	31		1039	
**PAS**				0.481
Yes	6		176	
No	28		1132	
**Tumor size (cm)**				0.266
≥ 46	19		605	
< 46	15		703	
**Tumor location**				0.094
Upper	13		304	
Middle	2		210	
Lower	14		656	
Diffuse	5		138	
**TNM stage**				0.000
I/ II	6		627	
III	28		681	
**Operation time (min)**				0.475
≥ 190	14		461	
< 190	20		847	
**BLDO (ml)**				
≥ 100	22		939	0.366
< 100	12		369	
**Resected LNs (*n***)				0.001
≥ 41	16		306	
< 41	18		1002	
Operation type				0.342
Total	15		685	
Subtotal	19		623	
**Type of Anastomosis Anastomosis Anastomosis**				0.332
Roux-en-Y	19		622	
B-I	9		538	
B-II	5		120	
Esophagogastric	1		28	
**Combined resection**				0.000
Yes	9		98	
No	25		1210	
**DTF**				0.000
Yes	6		36	
No	28		1272	
**PAI**				0.053
Yes	3		39	
No	31		1269	
**Chylous fistula**				0.073
Yes	3		42	
No	31		1266	
Hospital stay (days)	20.09 ± 9.67		13.86 ± 7.64	0.001

PAS: Previous abdominal surgery; PAI: Pure abdominal infection; DTF: Digestive tract fistula; BLDO: blood loss during the operation.

### Multivariate analysis of PH in the experimental group

The multivariate logistic regression analysis revealed that stage III tumors (*P* = 0.005, OR = 3.654), more than 41 lymph node excisions (*P* = 0.046, OR = 2.094), combined organs resection (*P* = 0.018, OR = 2.779) and postoperative digestive tract fistula (*P* < 0.001, OR = 6.898) were the independent risk factors of PH after LARG with D2 lymphadenectomy for primary gastric cancer. See Table 3.

**Table 3:** Multivariate analysis of PH

Variables	χ2	B	OR	95% CI	*P* value	Score
**TNM stage III**	7.894	1.296	3.654	1.480–9.025	0.005	1
**Resected LNs (*n***) ≥ 41	3.993	0.739	2.094	1.014–4.322	0.046	1
**Combined resection** Yes	5.630	1.022	2.779	1.195–6.463	0.018	1
**DTF** Yes	14.478	1.931	6.898	2.551–18.653	0.000	2

DTF: Digestive tract fistula.

### Risk gradation of PH and construction of the predictive scoring system

Because the differences in the regression coefficients ranged from 0.586 to 0.812, risk factors were simplified to the following weights based on their regression coefficients: a stage III tumor was denoted as 1 point, number of lymph node excisions ≥ 41 was denoted as 1 point, combined organ resection was denoted as 1 point and postoperative digestive tract fistula was denoted as 2 points. Based on these scores, patients were divided into low-risk (0 to 2 points) and high-risk (more than 3 points) groups. The low-risk group had 95.2% of the patients and the high-risk group had 4.8%. The hemorrhage rates were 2.0% and 13.8% in the low-risk and high-risk groups, respectively. Compared with the low-risk patients, the relative risk of high-risk patients was 8.04 (95% CI: 3.589–18.084, *p*<0.001). See Table 4.

**Table 4:** Risk gradation of PH

Risk gradation	Risk factor score	Case number (*n*, %)	Hemorrhage rate (*n*, %)	OR	95% CI	*P* value
**Low-risk** (1277) 95.2%	0	486(34.9%)	3 (0.6%)	1	/	/
	1	568 (42.3%)	11 (1.9%)			
	2	223 (16.6%)	11 (4.9%)			
**High-risk** (65) 4.8%	3	55 (4.1%)	6 (10.9%)	8.049	3.589–18.084	0.000
	4	7 (0.5%)	1 (14.3%)			
	5	3 (0.2%)	2 (66.7%)			

### The effectiveness test of the predictive scoring system

**Figure 1:**
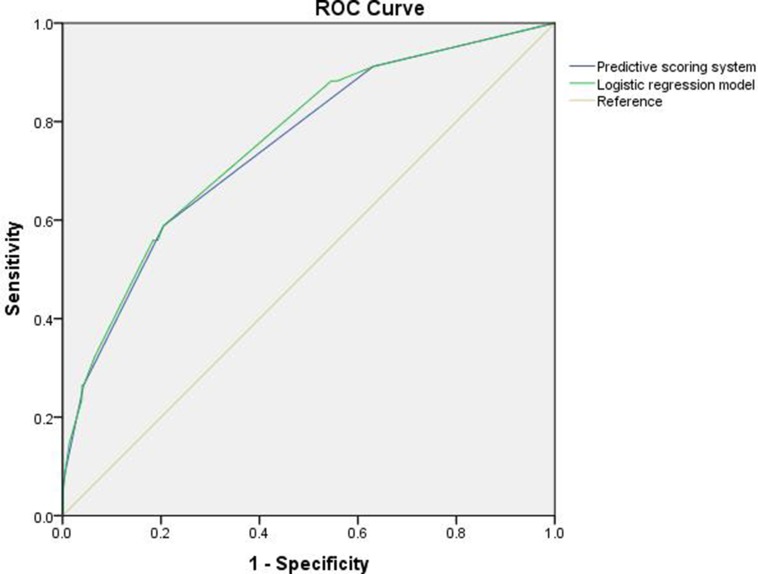
Receiver operating characteristic (ROC) curves for the logistic regression model and predictive scoring system for bleeding risk after LARG with D2 lymphadenectomy for PGC; the area under the ROC curve was 0.758 (0.674–0.842) for the logistic regression model, and 0.748 (0.663–0.834) for the simplified score system

The receiver operating characteristic (ROC) curve of this scoring system was calculated, and the area under the curve (AUC) was 0.748; see Figure 1. Detailed patient conditions in each group are shown in Table 5, which uses the scoring system to score and risk-stratify the validation group. The predictive ability of this scoring system was verified by determining whether there were differences between the predicted hemorrhage rate and the actual bleeding rate. We compared differences between high-risk and low-risk patients in the validation group with the actual rate of PH and the predicted rate of hemorrhage. There was no significant difference between them; the *p* values were 0.400 and 0.881 for the low-risk group and high-risk group, respectively. This indicated that the scoring system could accurately predict PH. See Table 5.

**Table 5:** Hemorrhage condition between the experimental group and verification group with the comparison between actual and predicted incidence of hemorrhage

Risk gradation	Score	CNEG (*n*, %)	IEG (*n*, %)	CNVG (*n*, %)	IVG (*n*, %)	*P* value
**Low-risk**		1277 (95.2%)	25 (2.0%)	434 (97.1%)	9 (2.1%)	0.881
	0	486 (34.9%)	3 (0.6%)	142 (31.8%)	1 (0.7%)	
	1	568 (42.3%)	11 (1.9%)	201 (45.0%)	3 (1.5%)	
	2	223 (16.6%)	11 (4.9%)	91 (20.4%)	5 (5.5%)	
**High-risk**		65(4.8%)	9(13.8%)	13(2.9%)	3(23.1%)	0.400
	3	55 (4.1%)	6 (10.9%)	11 (2.5%)	2 (18.2%)	
	4	7 (0.5%)	1 (14.3%)	2 (0.4%)	1 (50.0%)	
	5	3 (0.2%)	2 (66.7%)	0	/	

CNEG: Case number of experimental group; IEG: Incidence in experimental group.

CNVG: Case number of verification group; IVG: Incidence in verification group.

## DISCUSSION

Gastric cancer is one of the most common gastrointestinal malignancies. Its related disease mortality is second only to lung cancer [13–15]. To date, surgical resection is the primary intervention for GC [16]. With improvements in laparoscopic technology and surgical technique, laparoscopic surgery has become a common approach to surgical resection [14]. The number of applications for laparoscopic surgery has also increased. Although surgical treatment with R0 resection has become more common, postoperative complications remain a problem. Many centers reported that the incidence of the postoperative complications following LARG range from 11.6% to 18.7% [15, 16], and certain departments have reached incidence rates ranging from 24.9% to 42.6% [17, 18]. PH is one of the more serious complications. The literature reports that the incidence rate of PH following GC ranges from 0.6% to 3.3% and has a high mortality. [19, 20] In this study, the incidence rate of PH was 2.57%. Although we found a lower incidence of PH, it was still lethal. When patients present with PH, they tend to have longer hospital stays, more expensive treatments and a significantly increased risk of death [21]. Because of its severity, it is particularly important to effectively prevent PH following gastrectomy.

The risk factors for PH following gastrectomy are still controversial. Song et al. [21] believed that extensive lymph node excision and later tumor stages were the independent risk factors of PH. Park et al. [22] retrospectively analyzed clinical data from 5739 patients who suffered from PH following gastrectomy to treat PGC. Their results showed that 42% of patients with a PH complication had an early abdominal infection; another 62% had anastomotic or pancreatic leakage. They also reported that male gender and previous abdominal surgery were associated with PH following gastrectomy and have no relationship with neoadjuvant chemotherapy. Jeong et al. [23] reported that a higher BMI is associated with PH f.ollowing gastrectomy. The present study shows that patients with at least 41 lymph nodes excised, a later tumor TNM stage (stage III tumor), intraoperative combined organ resection and postoperative digestive tract leakage have a significantly higher risk of PH. Lymph nodes are located throughout the body near arteries and their branches. To achieve a radical cure, the surgeon must remove metastatic tissue from lymph nodes. This process requires stripping the lymph nodes and clearing nearby blood vessels. During lymphadenectomy, the high temperature of the ultrasonic knife may injure blood vessel walls and adjacent tissue, facilitating vascular damage and aneurysm formation, which can result in PH. In addition, combined organ resection usually expands the scope of the operation and makes wounds larger and PH more likely to occur. Our data also show that patients who suffered from postoperative gastrointestinal leakage have a high likelihood of PH occurring. The risk of PH in these patients is as much as 6.898 times greater than the risk of PH in patients without gastrointestinal leakage (95% CI: 2.551–18.653, *P* < 0.001). The body is stressed and has local inflammation after gastrectomy. Moreover, when patients suffer from postoperative gastrointestinal leakage, digestive juices seep into the abdominal cavity or the wound and erode blood vessels, resulting in arterial rupture or aneurysm formation. According to Japan's 14th edition of the gastric cancer treatment statute, [4], patients diagnosed with PGC at an advanced stage often need to expand scope during LARG with D2 lymphadenectomy. Surgeons will also resect other organs for the R0 resection when necessary. Therefore, patients with a later tumor TNM stage were usually in the high-risk group for PH. Once postoperative gastrointestinal leakage is found, surgeons should prepare for the possibility of subsequent hemorrhage and take strict precautions and appropriate perioperative interventions to reduce the risk of bleeding.

Although our study shows that PH following LARG with D2 lymphadenectomy for PGC was closely related to the above factors, the research about how to rapidly and accurately predict the risk of PH and take appropriate perioperative interventions when multiple risk factors are present was limited. This study established a scoring system based on the relevant risk factors. It combined characteristics of LARG and compared them using a logistic regression rating system to achieve a better match (area under the receiver-operating characteristic (ROC) curve (AUC) was 0.748). It also further compared the difference between the prediction ratios and the actual bleeding ratios of the validation group; there was no statistical significance, indicating that the scoring system for PH risks has good prediction ability. When this scoring system is used in a clinical management, surgeons should observe the patient's perioperative general condition dynamically and, at the same time, dynamically grade the patient according to the risk factors of hemorrhage to predict PH. For example, if a patient's preoperative assessment of the tumor is stage III, 1 point should be noted. If there is combined organ resection or more than 41 lymph nodes dissected, another point should be added. When the total score is ≥ 3, the patient should be categorized as high-risk, and the surgeon should closely monitor the patient's general condition. If digestive tract leakage occurs, 2 points are added and bleeding risk increases greatly. The surgeon should prepare for the possibility of hemorrhage and implement appropriate preventive measures, such as keeping the tube drainage unblocked, administering somatostatin to inhibit the secretion of digestive juices and so on. These measures promote the healing of leaks and reduce the risk of hemorrhage. Our study was based on large sample data from a single center, this scoring system can help young surgeons choose low bleeding risk patients to quickly and smoothly progress through the learning curve and promote the development and popularization of LARG.

There are some limitations of our study include the fact that the study is retrospective and non-randomized, and the sample size of patients with postoperative hemorrhage within 2 days (*n* = 18) and more than 1 week after surgery (*n* = 8) are small, thus there may be some selection bias if we evaluate separately according to the onset period of PH. In addition, the performance status for a few of patients was not recorded, which might result in some biases. So, this scoring system needs to be evaluated and validated by multicenter prospective trials.

In conclusion, this scoring system can effectively predict the risk of PH following LARG with D2 lymphadenectomy. It can also improve the risk awareness of PH after gastrectomy.
